# Electrochemical Flocculation Integrated Hydrogen Evolution Reaction of Fe@N‐Doped Carbon Nanotubes on Iron Foam for Ultralow Voltage Electrolysis in Neutral Media

**DOI:** 10.1002/advs.201901458

**Published:** 2019-07-22

**Authors:** Jiayuan Yu, Guixiang Li, Hui Liu, Lili Zeng, Lili Zhao, Jin Jia, Mingyuan Zhang, Weijia Zhou, Hong Liu, Yongyou Hu

**Affiliations:** ^1^ The Key Lab of Pollution Control and Ecosystem Restoration in Industry Clusters Ministry of Education Guangzhou Key Laboratory for Surface Chemistry of Energy Materials School of Environment and Energy South China University of Technology Guangzhou Higher Education Mega Centre Guangzhou 510006 P. R. China; ^2^ Shandong Collaborative Innovation Center of Technology and Equipements for Biological Diagnosis and Therapy Institute for Advanced Interdisciplinary Research (iAIR) University of Jinan Jinan 250022 P. R. China; ^3^ State Key Laboratory of Crystal Materials Shandong University Jinan 250100 P. R. China

**Keywords:** electrochemical flocculation, hydrogen evolution reaction, resource recovery, water purification, water splitting

## Abstract

Hydrogen (H_2_) production is a key step in solving the energy crisis in the future. Electrocatalytic water splitting suffers from sluggish anodic oxygen evolution reaction (OER) kinetics leading to low energy conversion efficiency. Herein, a strategy is presented that integrates anodic electrochemical flocculation with cathodic hydrogen production from water splitting in 0.5 m Na_2_SO_4_. Iron encapsulated in a nitrogen‐doped carbon nanotubes array on iron foam (Fe@N‐CNT/IF) is employed as an electrode for the hydrogen evolution reaction (HER), and the Fe@N‐CNT/IF possesses superior HER activity requiring an overpotential of 525 mV to achieve 10 mA cm^−2^, which is close to that of 20 wt% Pt/C. Benefiting from the lower oxidation potential of iron (*E*°_Fe/Fe2+_, 0.44 V) than that of OER (*E* ^0^_OH‐/O2_, 1.23 V), the cell voltage for integrated electrochemical flocculation and H_2_ production is significantly reduced by 1.31 V relative to overall water splitting to achieve 20 mA cm^−2^. More important, the production of electrochemical flocculation can be applied to water purification, because of the excellent adsorption capacity. Finally, metal–carbon electrocatalysts are prepared again by pyrolysis of flocculation adsorbents containing toxic heavy metals and organics. This result provides a new direction for designing a heterogeneous electrolysis system for energy conversion and environmental treatment applications.

## 1 Introduction

Energy crisis and environmental pollution are facing serious challenges in current social development. Hydrogen (H_2_), with zero pollution of its combustion product, has been considered as the cleanest energy resource to fulfil human need for future fuel applications.^1^ At present, the main method for hydrogen production is the steam reforming process of nonrenewable fossil energy, which accelerates depletion of fossil fuels and releases carbon dioxide. Electrochemical overall water splitting is an environmental‐friendly and high efficient technology by using electricity from sustainable and renewable energy resources (e.g., solar, wind, and wave) to produce high purity H_2_ gas.^2^ In fact, the scale‐up of hydrogen generation from overall water splitting has not been achieved, owing to the sluggish kinetic of the oxygen evolution reaction (OER).^3^ In addition, H_2_ and O_2_ were simultaneously generated during traditional water electrolysis, which must employ a chamber to separate the cathode and anode to avoid the potential explosion risk. Recently, a general electrolysis strategy for replacing OER with thermodynamically more favorable reactions has caused widespread attention.^4^ For example, the oxidation of alcohol or 5‐hydroxymethylfurfural on anode in alkaline media, in which required lower voltage to improve the energy conversion efficiency.[qv: 4b,e,f] However, the oxidation reactions of organic matters or biomass upgrade are usually valueless or add extra costs. Therefore, it is challenging for rational design of highly efficient, low‐cost, and safe anodic reaction integrated electrolysis system.

Electroflocculation is an effective process to remove pollutants in neutral wastewater, which was widely used in the field of environmental governance.^5^ The iron electrode was dissolved by applying positive potential for forming flocculant (hydroxy complex, polynuclear hydroxy complex, and hydroxide), which had strong adsorption capacity of pollutants (e.g., suspended particles, heavy metals, and organic matter). It is worth noting that the first step of the electroflocculation was iron oxidation and the standard potential of *E* ^0^(Fe/Fe^2+^) is 0.44 V at pH‐neutral condition (pH 7), which is much lower than the potential of OER *E* ^0^(OH^−^/O_2_, 1.23 V).^6^ Hence, it was reasonable for us to attempt to integrate the anodic electroflocculation process for water recovery with cathodic H_2_ generation to improve the energy conversion efficiency. However, the electroflocculation process need to be carried out in neutral media of Na_2_SO_4_ aqueous solution. As a result, it has been a daunting task to rational design of highly efficient and stable electrocatalysts for hydrogen evolution reaction (HER) in neutral electrolytes.

In the past decade, all kinds of molybdenum‐based, transition metal based and carbon based electrocatalysts have been reported with equivalent HER performances compare to state‐of‐the‐art Pt‐based materials in acidic or alkaline electrolyte.^7^ Recently, economic HER electrocatalysts based on earth‐abundant elements in neutral electrolyte (1 m phosphate buffered saline (PBS)) has attracted great interests because neutral electrolytes are environmentally‐benign and can efficiently extend the service life of electrodes.^8^ For example, Li and co‐workers reported a general promoting strategy for accelerating the Volmer steps to enhance HER, who designed and synthesized SiO_2_/PPy NTs‐CFs compound as high‐performance electrocatalyst for the HER in 1 m PBS.[qv: 8b] Sargent and co‐workers reported that Cu foam with weak hydrogen binding energy was modified with Ni atoms and CrO*_x_* cluster with strong hydrogen binding energies, which as electrode possessed high hydrogen production activity in 1 m PBS.[qv: 8c] However, the highly stable and active electrocatalysts in neutral media of Na_2_SO_4_ aqueous solution were rarely reported.

Herein, we reported an iron encapsulated in nitrogen‐doped carbon nanotubes array on iron foam (denoted as Fe@N‐CNT/IF) as effective HER electrode in 0.5 m Na_2_SO_4_, which required an overpotential of 525 mV to achieve the current density of 10 mA cm^−2^, which was close to state‐of‐the‐art 20 wt% Pt/C. In addition, a hybrid electrolyzer system of Fe@N‐CNT/IF(‐)//IF(+) composed of couple cathodic H_2_ production and anodic electroflocculation in neutral media. On anode, the flocculant produced from electroflocculation could efficiently absorb multiple pollutants, e.g., rhodamine B, methylene blue, methyl orange, ethylene diamine tetraacetic acid (EDTA)‐Ni, Cu^2+^, Cd^2+^, Cr^6+^, Co^2+^, and Ni^2+^. The Fe@N‐CNT/IF(‐)//IF(+) only required a cell voltage of ≈1.09 V to reach the current density of 20 mA cm^−2^ to realize simultaneously H_2_ production and water purification, which was much lower than that (2.40 V) of overall water splitting of Fe@N‐CNT/IF(‐)//RuO_2_/CC(+). It was more important that the obtained flocculent precipitate contained metals and organic elements on anode were reused and recycled to produce metal–carbon HER electrocatalysts. These results provided a new direction for the rational design of hybrid electrolyzer system with low operating voltage in neutral electrolyte, which was significant for energy conversion of hydrogen production and environmental protection of wastewater treatment.

## 2 Results and Discussion

**Figure 1:**
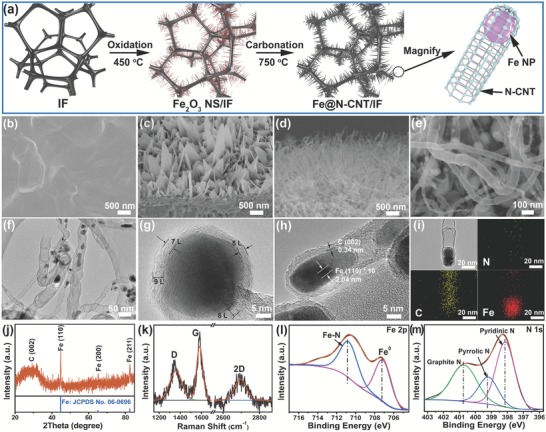
a) Schematic diagram of the synthesis process of Fe@N‐CNT/IF. FESEM images for b) IF, c) Fe_2_O_3_ NS/IF, d,e) Fe@N‐CNT/IF. f) TEM image, g,h) HRTEM images, and i) element mappings of N, C, and Fe for Fe@N‐CNT/IF. j) XRD pattern, k) Raman spectrum, XPS spectra in the l) Fe 2p and m) N 1s regions for Fe@N‐CNT/IF.

The fabrication processes of Fe@N‐CNT/IF were shown in **Figure** **1**a. First, Fe_2_O_3_ nanosheets array layer was in situ grown on iron foam (IF) by one step thermal treatment of precleaned IF in air (referred to Fe_2_O_3_ NS/IF, confirmed by X‐ray diffraction (XRD) in Figure S1 in the Supporting Information), then Fe_2_O_3_ NS/IF and dicyandiamide were put at a porcelain boat with cover together with two‐step heat treatment in a furnace under argon atmosphere flow. The dicyandiamide was acted as the carbon and nitrogen sources for nitrogen‐doped carbon nanotubes growth. Fe@N‐doped carbon nanotubes array was in situ growth on IF by following a “tip‐growth” mechanism (referred to Fe@N‐CNT/IF).^9^ During the pyrolysis, Fe_2_O_3_ NSs were reduced by carbon to form small size Fe metal nanoparticles, which were served as catalytic seeds for the tip growth of nitrogen‐doped carbon nanotubes (N‐CNT). As a result, the N‐CNT arrays with their apical domains encapsulating Fe nanoparticles were synthesized. As shown in Figure S2 (Supporting Information), the colors of IF, Fe_2_O_3_ NS/IF and Fe@N‐CNT/IF were silver white, reddish brown and black, respectively, implying the according phase transformation in the synthetic process.

Figure 1b showed the field‐emission scanning electron microscopy (FESEM) image of IF with smooth surface. As shown in Figure 1c, the FESEM image of Fe_2_O_3_ NS/IF with rough surface, confirming Fe_2_O_3_ NSs array covered on the surface of IF. After carbonization with dicyandiamide, the Fe_2_O_3_ NSs array format was converted to Fe@N‐CNT with broad diameters of 50–100 nm and lengths of 2–3 µm, which were confirmed by the cross‐section in Figure 1d and planform in Figure 1e. The perfect interface structure between Fe@N‐CNT and IF was good for electron transport and the multidimensional structure of Fe@N‐CNT providing more electrocatalytic area. The Fe_2_O_3_ NS was important to form Fe@N‐CNT, which provided more reaction area and active sites for the growth of carbon nanotube. In contrast, no N‐CNT was observed on the surface by using IF as precursor (Figure S3, Supporting Information). To further demonstrate the structure of Fe@N‐CNT, the according transmission electron microscope (TEM) images were shown in Figure 1f–h. Some nanoparticles with sizes of 5–20 nm were observed in the interior of nanotubes (Figure 1f), which was presumed as Fe nanoparticles. The high resolution transmission electron microscope (HRTEM) image in Figure 1g showed that the nanoparticle was wrapped by graphite wall and the thickness of carbon was ranged from 5 to 9 layers. HRTEM image taken from Figure 1h revealed well‐resolved lattice fringes with interplanar distances of 2.04 and 3.4 Å, which was no doubt corresponding to the (110) and (200) planes of the metallic Fe and graphite, respectively. The elemental mapping images of Fe@N‐CNT (Figure 1i) revealed the core–shell structure of uniform N‐doped carbon nanotube wrapped the Fe nanoparticle, which was on top of the carbon nanotubes.

The XRD pattern was used to identify the crystal structure of Fe@N‐CNT/IF as illustrated in Figure 1j. It was clearly shown that only metal Fe phases with the strong diffraction peaks at 44.6° (110), 65.0° (200), and 82.3° (211) were confirmed in Fe@N‐CNT/IF (JCPDS No. 06–0696, cubic, *a* = 2.8664 Å, *b* = 2.8664 Å, *c* = 2.8664 Å). No Fe_2_O_3_ phase was detected due to the strong reducing action of carbon derived from the decomposition of dicyandiamide. The weak XRD peaks around 26.6° were detected for carbon with low crystallinity. In order to verify the nature of carbon in the Fe@N‐CNT/IF, a Raman spectroscopy measurement was taken as shown in Figure 1k. The locations around at 1360, 1591, and 2700 cm^−1^ correspond to the graphite defect‐induced D band, the ordered graphitic structure of G band and 2D band, respectively.^10^ The high *I*_D_/*I*_G_ band intensity ratio of Fe@N‐CNT/IF indicated the generation of large amounts of defects, suggesting that a large amount of N atoms were doped in the graphitic carbon layers. Moreover, the shape and full width at half maximum (FWHM) of the second‐order graphitic structure of 2D band indicated that multilayers graphene was existed in the Fe@N‐CNT/IF,^10^ which was inconsistent with the result in Figure 1g. For a better insight of the chemical composition and electronic structure of the as‐synthesized Fe@N‐CNT/IF, X‐ray photoelectron spectroscopy (XPS) measurements were conducted. Figure S4a (Supporting Information) presented the full range of XPS spectrum, which clearly showed the elements of C, N, O, and Fe existing in the Fe@N‐CNT/IF. High‐resolution spectrum of Fe 2p level was displayed in Figure 1l, the photoelectron peak at 707.3 eV corresponding to the binding energy of Fe 2p_3/2_ was observed, which indicated the existence of Fe^0^. The other peak at 710.9 eV was corresponding to the nitrogen‐coordinated iron species.^11^ In Figure 1m, three peaks located at 397.2, 399.1, and 401.3 eV could be assigned to pyridinic N, pyrrolic N, and graphitic N, respectively. And the content of nitrogen element was detected about 4.86%. The XPS spectrum of C 1s (Figure S4b, Supporting Information) was fitted into two components, assigned to sp^2^ C–C bonding from surface adventitious carbon at 284.5 eV and C–N at 285.4 eV, confirming the N doping into carbon.

**Figure 2:**
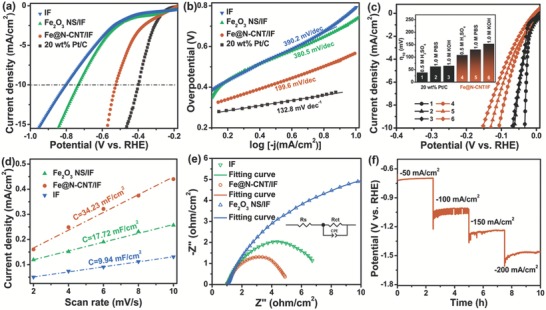
a) Polarization curves and b) Tafel slope of IF, Fe_2_O_3_ NS/IF, Fe@N‐CNT/IF, and 20 wt% Pt/C in 0.5 m Na_2_SO_4_, respectively. c) Polarization curves for HER in traditional all‐pH electrolytes (0.5 M H_2_SO_4_, 1.0 M PBS and 1.0 M KOH) of Fe@N‐CNT/IF and 20 wt% Pt/C. d) The double‐layer charging currents at −0.2 V vs RHE as a function of scan rate and e) electrochemical impedance spectroscopy (EIS) Nyquist plots at −0.6 V versus RHE recorded of IF, Fe_2_O_3_ NS/IF, and Fe@N‐CNT/IF, respectively. f) Multicurrent process of Fe@N‐CNT/IF. The current density started from −50 to −200 mA cm^−2^, with an increment of −50 mA cm^−2^ per 2.5 h.

The electrocatalytic HER performances for IF, Fe_2_O_3_ NS/IF, Fe@N‐CNT/IF, and 20 wt% Pt/C were assessed in 0.5 m Na_2_SO_4_ solution. The polarization curves illustrated in **Figure** **2**a showed increasing HER activities in the order of IF < Fe_2_O_3_ NS/IF < Fe@N‐CNT/IF < 20 wt% Pt/C. The Fe@N‐CNT/IF exhibited superior HER activity in 0.5 m Na_2_SO_4_ solution and required an overpotential of 525 mV to achieve 10 mA cm^−2^, which was smaller than that of bare IF (802 mV) and Fe_2_O_3_ NS/IF (734 mV) but larger than that of 20 wt% Pt/C (399 mV). The according Tafel slopes were also compared in Figure 2b, which were obtained via the η = *a* +*b*log|*j*|. The Tafel slope of Fe@N‐CNT/IF was 199.6 mV dec^−1^, which was lower than those of other samples, including IF (380.5 mV dec^−1^) and Fe_2_O_3_ NS/IF (390.2 mV dec^−1^), yet larger than that of 20 wt% Pt/C (132.8 mV dec^−1^). On the basis of the Volmer–Heyrovsky HER mechanism, the rate‐limiting step was the electrochemical discharge process for all the samples. In fact, the HER activity of reported electrocatalysts were usually evaluated in 0.5 m H_2_SO_4_, 1.0 m PBS, and 1.0 m KOH, respectively.[qv: 2a,7b,12] No reported electrocatalysts were evaluated in Na_2_SO_4_ electrolyte for HER due to slow reaction kinetics. However, the Na_2_SO_4_ electrolyte was necessary for anode electroflocculation to produce iron flocculation, which was an important way to treat waste water in the environmental field. Therefore, for a fair comparison with the reported electrocatalysts, the obtained Fe@N‐CNT/IF electrocatalyst was also tested in 0.5 m H_2_SO_4_, 1.0 m PBS, and 1.0 m KOH, respectively, which also possessed the excellent HER performance (Figure 2c). The remarkable HER performance of Fe@N‐CNT/IF was compared to other highly active HER non‐noble electrocatalysts (Table S1, Supporting Information), which was better than, or at least comparable to, those of non‐noble electrocatalysts, implying the high intrinsic catalytic activity of Fe@N‐CNT/IF.

Typically, the catalytic current density of HER was related to the number of catalytically active site of electrocatalysts and could be measured by the double layer capacitance (Figure 2d; Figure S5, Supporting Information). The electrochemically active surface area value of Fe@N‐CNT/IF was 34.23 mF cm^−2^, which was larger than that of IF (9.94 mF cm^−2^) and Fe_2_O_3_ NS/IF (17.72 mF cm^−2^), respectively. Generally, the larger electrochemical surface area (ECSA) corresponded to more catalytic sites contributing to higher current density. Even after being corrected by ECSA, the HER performance of Fe@N‐CNT/IF still possessed the best HER performance in 0.5 m Na_2_SO_4_ solution (Figure S6, Supporting Information), implying that the enhanced HER activity was not only from the increased electrochemical surface area but also from improvement of essential electrocatalytic activity. Electrochemical impedance spectroscopy (EIS) was also performed in Figure 2e to study the electrocatalytic kinetics in HER. As we all know, the smaller charge transfer resistance (*R*_ct_) corresponded to the faster reaction rate, which was obtained from the semicircle in the low frequency zone. The *R*_ct_ of Fe@N‐CNT/IF was smaller than those of IF and Fe_2_O_3_ NS/IF at the potential of −0.6 V versus RHE, suggesting that Fe@N‐CNT/IF had a faster electron transfer rate at the electrocatalyst/electrolyte interface. Figure S7 (Supporting Information) also depicted the Nyquist plots acquired under various overpotentials for Fe@N‐CNT/IF electrocatalysts. It could be seen that the *R*_ct_ of Fe@N‐CNT/IF decreased significantly with the increasing overpotentials, from 27.5 Ω cm^−2^ at −560 mV to 5.25 Ω cm^−2^ at −760 mV, verified by the obvious contraction of the semicircle diameter, suggesting the favorable charge transfer at the interface between electrolyte and electrode.

The stability of the electrocatalyst is also crucial, as a significant judgment of catalytic activity for HER. To evaluate the stability of the Fe@N‐CNT/IF, the electrode was operated at different current density to obtain the time‐dependent potentials. As shown in Figure 2f, the voltage–time curve of Fe@N‐CNT/IF was performed over 10 h at a multicurrent‐density. The nearly invariant voltage of constant −50, −100, −150, and −200 mA cm^−2^ indicated the good catalytic durability of the Fe@N‐CNT/IF in 0.5 m Na_2_SO_4_. After *v–t* testing for 10 h, no significant change in HER electrochemical performance (Figures S8–S10, Supporting Information), morphology (Figure S11, Supporting Information), and crystal structure (Figure S12, Supporting Information) of Fe@N‐CNT/IF were observed, which confirmed that the structure stability as well as catalytic stability.

**Figure 3:**
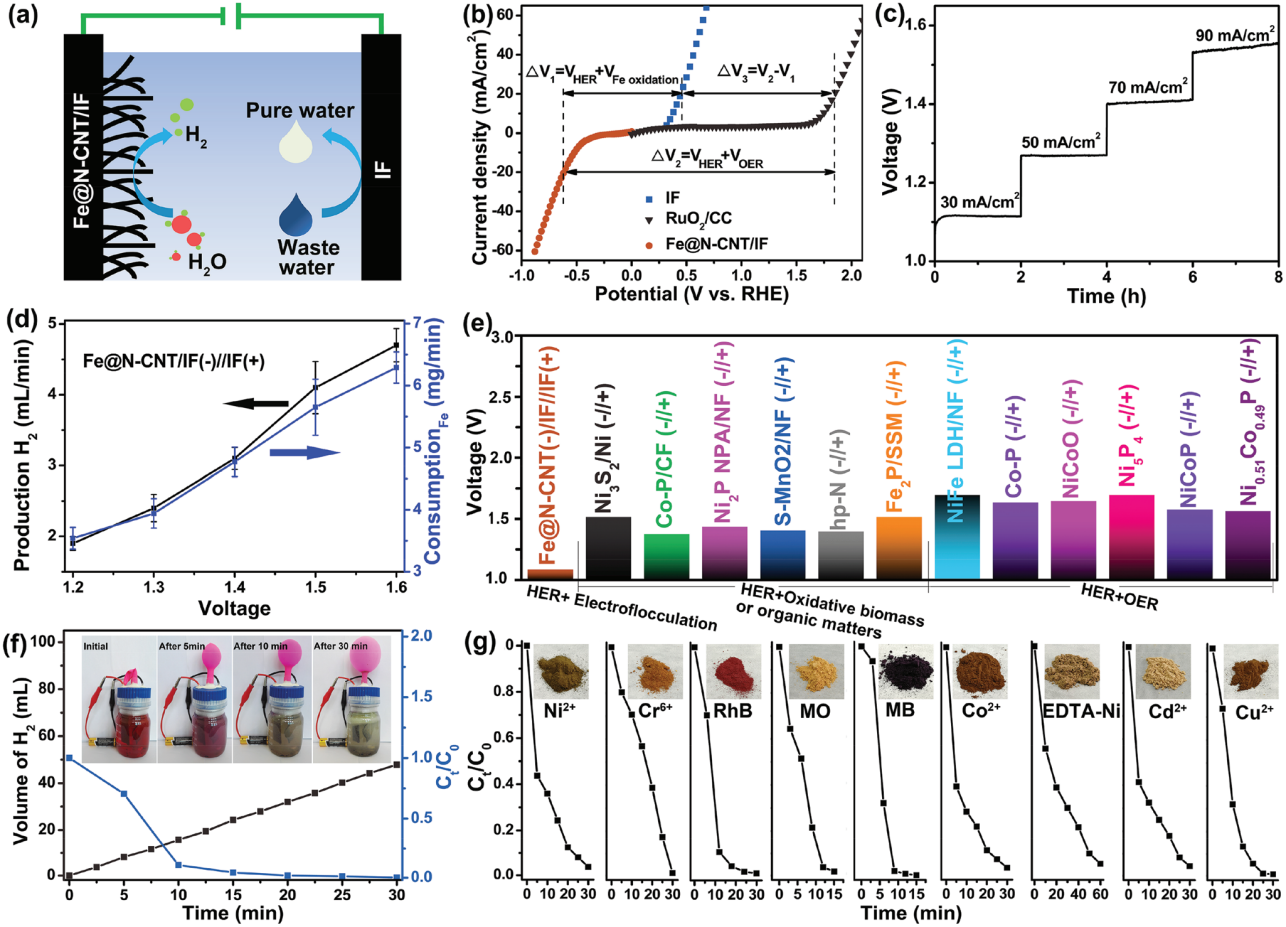
a) Concept of the coupled systems of electrolytic hydrogen evolution on cathode and water purification on anode. b) Polarization curves of Fe@N‐CNT/IF (cathode for HER), IF (anode for oxidation of Fe), and RuO_2_ loaded on carbon cloth (anode for OER) in 0.5 m Na_2_SO_4_. c) Multicurrent process of Fe@N‐CNT/IF(‐)//IF(+). The current density started at 30 mA cm^−2^ and ended at 90 mA cm^−2^, with an increment of 20 mA cm^−2^ per 2 h. d) The relationship between hydrogen production and amount of dissolved iron. e) Comparison of the reported electrolyzers. f) GC‐measured H_2_ quantity and RhB removal rate for the Fe@N‐CNT/IF(‐)//IF(+) coupled system driving by a commercial 1.5 V AA battery. The insert optical photos of Fe@N‐CNT/IF(‐)//IF(+). g) The purification performance of different pollutants by Fe@N‐CNT/IF(‐)//IF(+) coupled system. Insets were the photos of according anode flocculation precipitation powder.

Higher energy conversion efficiency of electrolysis of water encouraged us to employ more effective anodic reaction to replace kinetically slow OER. A coupled and a home‐made electrolyzer system was consisted of HER via Fe@N‐CNT/IF as cathode to produce H_2_ and electrocoagulation via IF as anode to simultaneously recover waste water (schematic in **Figure** **3**a). The standard iron oxidation potential of *E* (Fe/Fe^2+^) is 0.44 V at pH‐neutral condition (pH 7), which is much lower than the potential of *E* ^0^ (OH^−^/O_2_, 1.23 V).[qv: 7d] In order to confirm the mechanism, the comparison between oxidation reaction of IF and OER of commercial RuO_2_ loaded on CC with same loading amount of 1.2 mg cm^−2^ was carried out as shown in Figure 3b. The RuO_2_ exhibited sluggishness activity for OER in Na_2_SO_4_ solution and required an overpotential of 1.85 V versus RHE to achieve the current density of 10 mA cm^−2^. Inspiring, the oxidation of Fe reaction only required an overpotential of 0.46 V versus RHE to achieve the current density of 10 mA cm^−2^.

For comparison of the energy conversion efficiency, two types of electrolyzers, were established in 0.5 m Na_2_SO_4_ solution, Fe@N‐CNT/IF(‐)//RuO_2_/CC(+) for overall water splitting and Fe@N‐CNT/IF(‐)//IF(+) for HER and oxidation of iron. In the hybridization electrolyzer of Fe@N‐CNT/IF(‐)//IF(+), the electrolysis voltage was significantly reduced to 1.09 V to achieve 20 mA cm^−2^, which was much lower than that (2.40 V) of Fe@N‐CNT/IF(‐)//RuO_2_/CC(+) as shown in Figure 3b and Figure S13 in the Supporting Information. As shown in Figure 3c, the voltage–time curves were performed over 8 h at a multicurrent‐density in 0.5 m Na_2_SO_4_ to evaluate the operational stability of the Fe@N‐CNT/IF(‐)//IF(+). The nearly invariant voltage of constant from 30 to 90 mA cm^−2^ indicated the good catalytic durability before the fracture of electrode. The IF as anode was gradually consumed to form iron flocculation in this hybridization electrolyzer, and the relationship between hydrogen production amount of Fe@N‐CNT/IF on cathode and dissolved iron amount of IF anode was shown in Figure 3d. The calculated result confirmed that the Fe anode lose two electrons to form one Fe^2+^ ion, which reduced two H^+^ ions to form one H_2_ molecule. No any bubbles observed on the surface of IF anode also confirmed that only iron oxidation reaction to generate ferrous ions occurred not OER on IF anode (Figure S14a, Supporting Information). In order to show the remarkable superiorities of Fe@N‐CNT/IF(‐)//IF(+) with ultralow electrolysis voltage in neutral media, a detailed comparison of two‐electrode systems, including overall water splitting and HER integrated oxidative biomass, was provided in Figure 3e and Table S2 in the Supporting Information. It was worth noting that, Fe@N‐CNT/IF(‐)//IF(+) possessed the lowest electrolysis voltage among all reported results, even though HER reaction kinetics were slow in Na_2_SO_4_ aqueous solution. The Fe@N‐CNT/IF(‐)//IF(+) electrolyzer not only reduced the electrolytic voltage for hydrogen evolution, but also had the function of water purification. The flocculation (a hydroxy complexes, polynuclear hydroxy complexes, and hydroxides) was generated by a series of hydrolysis, polymerization and oxidation of Fe ions from IF anode in Na_2_SO_4_ solution (Figure S14b, Supporting Information), which was detected rich in functional groups (Figure S15, Supporting Information) and had strong adsorption capacity of pollutants. Because of ultralow voltage electrolysis of 1.09 V in neutral media, the Fe@N‐CNT/IF(‐)//IF(+) electrolyzer can be driven by a commercial 1.5 V AA battery for hydrogen production and water purification (Figure 3f). The gas generated from the coupled system was determined to be H_2_ by gas chromatographic measurements and the generation rate was confirmed to be 4 mL min^−1^ at 1.5 V. At the same time, the color of the electrolyte containing red Rhodamine B (RhB) gradually became colorless, due to the effective adsorption of RhB by flocculation and the removal rate was 99.2% in first 10 min. The simultaneous dynamic process of generated H_2_ collected in balloon and waste water purification were observed by the inset photos of the actual home‐made coupled system device in Figure 3f. Furthermore, the anodic flocculation can remove all kinds of organic pollutants and heavy metal ions as shown in Figure 3g. The removal rates of pollutants were 95.7% for Cd^2+^, 96.6% for Co^2+^, 96.6% for Ni^2+^, 96.3% for Cu^2+^, 98.8% for Cr^6+^, 94.5% for EDTA‐Ni, 98.2% for MO, and 99.3% for RhB in 30 min. From the inset photos in Figure 3g, the obtained flocculent precipitates possessed different colors, which also confirmed the successful adsorption of organic pollutants and heavy metal ions, respectively.

**Figure 4:**
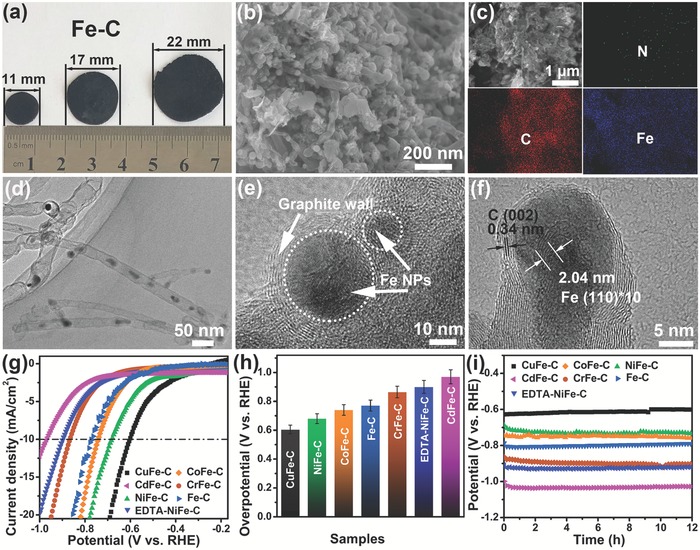
a) The photos, b,c) SEM images, and C, N and Fe mappings, d) TEM image, e,f) HRTEM images of electrode composed of Fe–C. g) Polarization curves for HER in 0.5 m Na_2_SO_4_ at GC electrode modified by Fe‐C and MFe‐C (M = Cr, Ni, Co, Cu, Cd, EDTA‐Ni), respectively. h) Comparison of the overpotentials of different samples at a current density of 10 mA cm^−2^. i) The potential–time plots at the applied current density of 10 mA cm^−2^ for 12 h.

At last, the operating cost of Fe@N‐CNT/IF(‐)//IF(+) electrolyzer was evaluated in terms of energy and environment, respectively. As for new energy production, the power consumption cost of Fe@N‐CNT/IF(‐)//IF(+) save about 65% cost of Fe@N‐CNT/IF(‐)//RuO_2_/CC(+) due to lower working voltages (1.09 vs 2.40 V) with same hydrogen production output. However, the consumption of iron from anode also increased operating cost of Fe@N‐CNT/IF(‐)//IF(+), which was calculated in the Supporting Information. The cost reduction by voltage reduction and increase by iron consumption was basically offset. The significant advantages of Fe@N‐CNT/IF(‐)//IF(+) were also summarized. First, the IF anode can be prepared from low‐cost scrap iron, which was much cheaper than that of RuO_2_ electrocatalyst. Second, the no production of oxygen in Fe@N‐CNT/IF(‐)//IF(+) avoided mixing oxygen and hydrogen, which simplified the structure of the electrolytic cell and improved the safety of the electrolyzer. Furthermore, the anodic flocculation produced from Fe@N‐CNT/IF(‐)//IF(+) can remove all kinds of organic pollutants and heavy metal ions, which applied to the sewage treatment in environmental field. As for environmental protection, the high value‐added hydrogen produced from Fe@N‐CNT/IF(‐)//IF(+) can compensate for the cost of sewage treatment, which encourage enterprises to take an active part in environmental protection and governance. Furthermore, the precipitates containing metal ions and organic matters generated from anodic flocculation process can be recycled as metal@carbon electrocatalysts for HER, which was confirmed in **Figure** **4**.

The precipitates generated from waste water treatment process containing metal ions and organic matters were usually considered as solid waste.^13^ However, the carbon and metal were two kinds of valuable elements in the field of electrocatalysis, which improved conductivity and produced new catalytic active sites, respectively.[qv: 7h,14] In this work, the obtained precipitation from water purification by electrocoagulation has potential utilization value due the rich content of metals, N and C elements. For example, the iron flocculation with adsorbed MB precipitate was easily constructed as wafer shape with diameters of 11, 17, and 22 mm by compression methods (shown in Figure S16 in the Supporting Information), then under calcination at 750 °C in Ar atmosphere. In the calcination process, MB served as C and N sources and iron flocculation as metal source synthesized Fe–C nanomaterial. As shown in Figure 4a, the obtained plates remained intact except for a little warping at the edge, which could be directly employed as bulk electrode. Abundant carbon nanotubes with diameters of 50–300 nm were observed on the surface of electrode by FESEM (Figure 4b). In addition, the uniform distribution of C, N, and Fe elements on the electrode surface was further confirmed by elemental mapping in Figure 4c. The TEM image in Figure 4d showed that some nanoparticles were observed in the interior of nanotubes, which was presumed as Fe nanoparticles. HRTEM images taken from Figure 4e,f revealed the core–shell structure and well‐resolved lattice fringes with interplanar distances of 2.04 and 3.4 Å, which corresponding to the (110) and (200) planes of the metallic Fe and graphite, respectively.

In general, it was feasible to use discard precipitates to obtain metal–carbon electrocatalysts. The iron flocculation adsorbed MB with all kinds of metals ions including Cu ions, Ni ions, Cr ions, Co ions, EDTA‐Ni, and Cd ions were used to synthesize multimetal carbon hybrids by similar calcination process with that of Fe@N‐CNT without dicyandiamide. The crystal components and morphologies of obtained CuFe‐C, NiFe‐C, CrFe‐C, CoFe‐C, EDTA‐NiFe‐C, and CdFe‐C compounds were determined by XRD (Figure S17, Supporting Information) and SEM (Figure S18, Supporting Information), respectively. The HER performances of the Fe‐C and MFe‐C (M = Cr, Ni, Co, Cu, Cd, EDTA‐Ni) electrocatalysts in 0.5 m Na_2_SO_4_ solution were shown in Figure 4g,h. The overpotentials at a current density of 10 mA cm^−2^ increased in the order of CuFe‐C (605 mV), NiFe‐C (680 mV), CoFe‐C (740 mV), Fe‐C (771 mV), CrFe‐C (865 mV), EDTA‐NiFe‐C (901 mV), and CdFe‐C (970 mV). The Tafel slope of CuFe‐C was 277.8 mV dec^−1^, which was lower than those of other samples, including NiFe‐C (296.5 mV dec^−1^), CoFe‐C (305.4 mV dec^−1^), Fe‐C (306.9 mV dec^−1^), CrFe‐C (315.1 mV dec^−1^), EDTA‐NiFe‐C (318.6 mV dec^−1^), and CdFe‐C (327.7 mV dec^−1^) as shown in Figure S19 in the Supporting Information. All the samples above proceeded through a Volmer–Heyrovsky HER mechanism with the rate‐limiting step of the electrochemical discharge process. The voltage–time plots at the applied current density of 10 mA cm^−2^ showed a stable potential for 12 h (Figure 4i), indicating long‐term catalytic viability of obtained metal–carbon electrocatalysts under operating conditions.

## 3 Conclusion

In conclusion, we have presented a novel strategy for integrating electrochemistry water splitting for hydrogen production with electrocoagulation for water purification in neutral media. In the present case, hydrogen produced at the cathode electrocatalyzed by Fe@N‐CNT/IF, while simultaneous oxidation of IF and generated flocculant for absorbing contaminants. Due to the more favorable thermodynamics of iron oxidation than that of oxygen evolution reaction, the electrolytic cell voltage to reach the current density of 20 mA cm^−2^ for hydrogen generation was lower to ≈1.09 V than that of overall water splitting (2.40 V). Additionally, the flocculant generated at the anode with strong adsorption capacity of organics and heavy metals. More important, metal–carbon composites were obtained by controlled pyrolysis of recyclable flocculation adsorbents containing a variety of heavy metals and organics. We forecast that this strategy is very promising and practical for future new energy conversion and environmental treatment applications.

## Conflict of Interest

The authors declare no conflict of interest.

## Supporting information

Supplementary
