# Supplementary material for: Electrochemical Flocculation Integrated Hydrogen Evolution Reaction of Fe@N‐Doped Carbon Nanotubes on Iron Foam for Ultralow Voltage Electrolysis in Neutral Media
Source: Adv Sci (Weinh). 2019 Jul 22;6(18):1901458. doi: 10.1002/advs.201901458 (PMC6755524; doi:10.1002/advs.201901458)
Supplement: Supplementary file 1 — Supplementary [file ADVS-6-1901458-s001.pdf]

Copyright WILEY-VCH Verlag GmbH & Co. KGaA, 69469 Weinheim, Germany, 2019.

ADVANCED SCIENCE Open Access

Supporting Information

for *Adv. Sci.,* DOI: 10.1002/advs.201901458

Electrochemical Flocculation Integrated Hydrogen Evolution Reaction of Fe@N-Doped Carbon Nanotubes on Iron Foam for Ultralow Voltage Electrolysis in Neutral Media

Jiayuan Yu, Guixiang Li, Hui Liu, Lili Zeng, Lili Zhao, Jin Jia, Mingyuan Zhang, Weijia Zhou, Hong Liu, and Yongyou Hu**

Electrochemical Flocculation Integrated Hydrogen Evolution Reaction of Fe@N-doped Carbon Nanotubes on Iron Foam for Ultra-low Voltage Electrolysis in Neutral Media

Jiayuan Yu, Guixiang Li, Hui Liu, Lili Zeng, Lili Zhao, Jin Jia, Mingyuan Zhang, Weijia Zhou, Hong Liu, Yongyou Hu**

Dr. J. Yu, G. Li, H. Liu, L. Zeng, Dr. M. Zhang, Prof. W. Zhou, Prof. Y. Hu
The Key Lab of Pollution Control and Ecosystem Restoration in Industry Clusters, Ministry of Education, Guangzhou Key Laboratory for Surface Chemistry of Energy Materials, School of Environment and Energy, South China University of Technology, Guangzhou Higher Education Mega Centre, Guangzhou, 510006, P. R. China.
E-mail: eszhouwj@scut.edu.cn (W. Zhou), ppyyhu@scut.edu.cn (Y. Hu)

Dr. J. Yu, Dr. L. Zhao, Dr. J. Jia, Prof. W. Zhou, Prof. H. Liu
Shandong Collaborative Innovation Center of Technology and Equipements for Biological Diagnosis and Therapy, Institute for Advanced Interdisciplinary Research (iAIR), University of Jinan, Jinan, 250022, P. R. China.

Prof. H. Liu
State Key Laboratory of Crystal Materials, Shandong University, Jinan, 250100, P. R. China.

Corresponding Authors
* E-mail: eszhouwj@scut.edu.cn (W. Zhou).
* E-mail: ppyyhu@scut.edu.cn (Y. Hu).

Author Contributions

[#] (J. Yu and G. Li) These authors contributed equally to this work.

Experimental

Material synthesis

Chemicals

All reagents were of analytical grade and used without further purification. Iron foam (10 cm ×10 cm × 0.1 cm) was purchased from Suzhou Taili foam metal material Co., Ltd (China). Dicyandiamide ($C_2H_4N_4$), potassium hydroxide (KOH), dibasic sodium phosphate (Na_2HPO_4), sodium dihydrogen phosphate (NaH_2PO_4), ethanol (C_2H_5OH), hydrochloric acid (HCl), sulfuric acid (H_2SO_4), ruthenium oxide (RuO_2) and 20 wt% Pt/C were purchased from Sinopharm Chemical Reagents Beijing Co.. Rhodamine B ($C_{28}H_{31}ClN_2O_3$), methylene blue ($C_{16}H_{18}ClN_3S$), methyl orange ($C_{14}H_{14}N_3SO_3Na$), potassium dichromate ($K_2Cr_2O_7$), cupric sulfate ($CuSO_4$), nickel sulfate ($NiSO_4 \cdot 6H_2O$), cobaltous nitrate ($Co(NO_3)_2 \cdot 6H_2O$), cadmium nitrate ($Cd(NO_3)_2$), ethylenediaminetetraacetic acid ($C_{10}H_{16}N_2O_8$) were purchased from Tianjin Damao Chemical Reagent Factory. Nafion solution (~5 wt% in a mixture of lower aliphatic alcohols and water) was purchased from Sigma-Aldrich Shanghai Co.. Deionized water was supplied with a Barnstead Nanopure Water System (18.2 MΩ·cm) and used throughout the experiment. Argon gas and nitrogen gas were obtained from Guangzhou YIGAS Gases Co., Ltd..

Synthesis of Fe_2O_3 Nanosheets Array on Iron Foam (Fe_2O_3 NS/IF)

The synthesis process of Fe_2O_3 NS/IF was according to the literature with a modification.[1] In a typical synthesis process, IF of 0.1 cm in thickness were cut into specimens of 2 cm × 4 cm, then sonicated in ethanol, diluted hydrochloric acid (0.01 M) and deionized water for 15 min, respectively. After drying in a nitrogen flow, the IF was thermally annealed at 450 °C for 4 h with a heating rate of 2 °C/min in air and cooled down naturally. The obtained red-brown product was denoted as Fe_2O_3 NS/IF.

Synthesis of Fe Nanoparticles Encapsulated into N-Doped Carbon Nanotubes Array on Iron Foam (Fe@N-CNT/IF)

A piece of Fe_2O_3 NS/IF and dicyanamide were put at a porcelain boat with cover. The weight ratio for Fe_2O_3 NS/IF to dicyandiamide was 1:10. Subsequently, the sample was heated at 450 °C for 2 h, then at 750 °C for 2 h under Ar atmosphere. The obtained black product was denoted as Fe@N-CNT/IF. The mass loading of Fe@N-CNT is ~1.2 mg/cm^2, which was dissolved to remove IF by 3 M HCl.

Synthesis of Metal–Carbon Hybrids Electrode

In a typical experiment, the Fe-based flocculant adsorbed of methylene blue or heavy metal (denoted as Fe-MB or Fe-M (M=Cr, Co, Ni, Cu, EDTA-Ni or Cd)) was firstly filtered out and dried. Coequal mass mixture of Fe-MB and Fe-M were put into a ceramic boat and calcined at 750 °C for 2 h in an Ar atmosphere to synthesize multimetal carbon hybrids, which were denoted as MFe–C (M=Cr, Co, Ni, Cu, EDTA-Ni, Cd). Fe-MB was calcined at 750 °C for 2 h in an Ar atmosphere to obtain monometal carbon hybrids (Fe–C).

Characterization

The morphologies of the obtained samples were characterized by field-emission scanning electron microscopy (FESEM, HITACHI UHR FE-SEM, SU8010). Transmission electron microscopy (TEM) was performed using a JOEL JEM 2100F microscope. Powder X-ray diffraction (XRD) patterns of the samples were recorded using a Bruke D8 Advance powder X-ray diffractometer with Cu Kα (λ = 0.15406 nm) radiation. X-ray photoelectron spectroscopy (XPS) was performed using a PHI X-tools instrument (ULVAC-PHI). Raman spectra were obtained by a LabRAM HR800 spectrometer (Horiba Jobin Yvon) equipped with an Ar laser (wavelength = 514.5 nm) and a long working distance 50× objective lens. Fourier transform infrared spectroscopy (FTIR) of all the samples were recorded via an IR Affinity-1 FTIR spectrometer (Shimadzu, Japan).

Electrochemical Measurements

Hydrogen evolution reaction catalytic activities were tested using an electrochemical workstation (CHI 760E, CH Instruments, Inc.) in a standard three-electrode system in 0.5 M Na_2SO_4 electrolyte, using as-prepared electrode, a saturated calomel electrode (Hg/Hg_2Cl_2 in a saturated KCl solution) and graphite rod electrode (diameter: 6 mm, length: 100 mm) as the working, reference and counter electrode, respectively. For preparation of 20 wt% Pt/C and RuO_2 electrode, commercial 20 wt% Pt/C or RuO_2 was well-dispersed in diluted Nafion alcohol solution (1 mL of ethanol and 50 μL of Nafion) to form a homogeneous suspension. Then, the suspension was drop cast onto carbon cloth (CC) and dried at room temperature and the loading amount was about 1.2 mg/cm^2. Electrochemical activity of the samples (IF, Fe_2O_3 NS/IF, Fe@N-CNT/IF, metal–carbon hybrids electrode and 20 wt% Pt/C electrodes) were studied via linear sweep voltammetry (LSV) curves with the potential from 0 to -1 V vs. RHE

(E(RHE)=E(Hg/Hg_2Cl_2, saturated KCl)-0.059×pH) at a potential sweep rate of 2 mV/s. Electrochemical impedance spectroscopy (EIS) was performed with an amplitude of 10 mV and a frequency range of 100 kHz to 0.01 Hz. The main arc in the EIS spectra was fitted using a simplified Randles equivalent circuit, which consisted of a resistance (R_s), a charge-transfer resistance (R_{ct}, interface electrocatalytic reaction between the electrode and electrolyte) and a constant-phase element, and the fitting parameters were estimated using the Levenberg-Marquardt minimization procedure. CV was used to probe the electrochemical double layer capacitance at non-faradaic potentials for estimating the effective electrode surface area. The current-time responses were monitored by chronoamperometric measurements for 10 h at different current density. The produced hydrogen was analyzed by an online gas chromatograph equipped with a thermal conductivity detector (HXSP-GC950, China, TCD, nitrogen as a carrier gas and 5 Å molecular sieve column).

Electrochemical Flocculation Adsorption Measurements

The electroflocculation measurement was tested in a standard two-electrode system in 100 mL 0.5 M Na_2SO_4 with 100 mg heavy metal ions or organic pollutants. The initial concentration of pollutants (methyl orange (MO), methylene blue (MB), rhodamine B (RhB), Cu^{2+}, Co^{2+}, Cd^{6+}, Ni^{2+}, Cd^{2+}) was 1 g/L and that of EDTA-Ni was 10 mM. For two-electrode electrolysis, Fe@N-CNT/IF and IF were employed as cathode and anode, respectively. The electroflocculation was started by chronoamperometric measurement at the voltage of 1.5 V. During electroflocculation experiments, the solution was continuously stirred at a rotate speed of 200 rpm. In a regular interval, 2 mL of the mixture was filtered to measure the concentration of contaminants in the simulated wastewater. The concentrations of RhB, MB, MO or EDTA-Ni were determined by UV-Vis absorption spectrometer (UV-6100, Metash). The concentrations of heavy metal ions (Cu^{2+}, Cd^{2+}, Cr^{6+}, Co^{2+} and Ni^{2+}) were determined by inductively coupled plasma atomic emission spectrometry (ICP-AES).

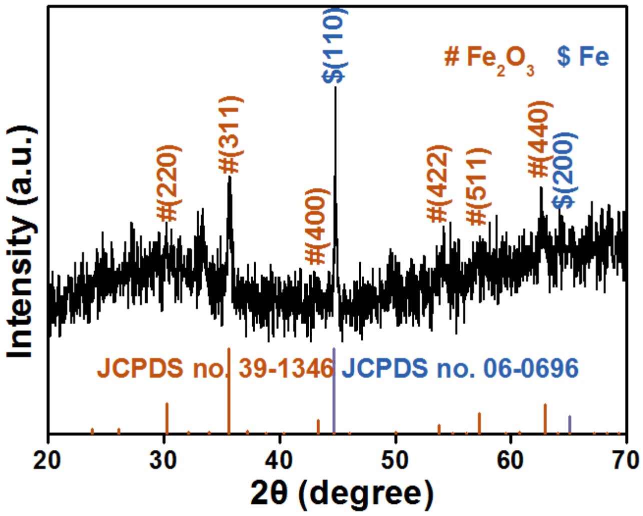

Figure S1 XRD pattern of Fe_2O_3 NS/IF.

The X-ray diffraction (XRD) pattern was used to identify the crystal structure of Fe_2O_3 NS/IF as shown in **Figure S1**. It is clearly show that Fe_2O_3 phases with the strong diffraction peaks at 30.2°, 35.6°, 43.2°, 53.7°, 57.2° and 62.9° were observed in Fe_2O_3 NS/IF corresponding to (220), (311), (400), (422), (511), (440) and (311) crystal planes of Fe_2O_3 phase (JCPDS No. 39-1346).

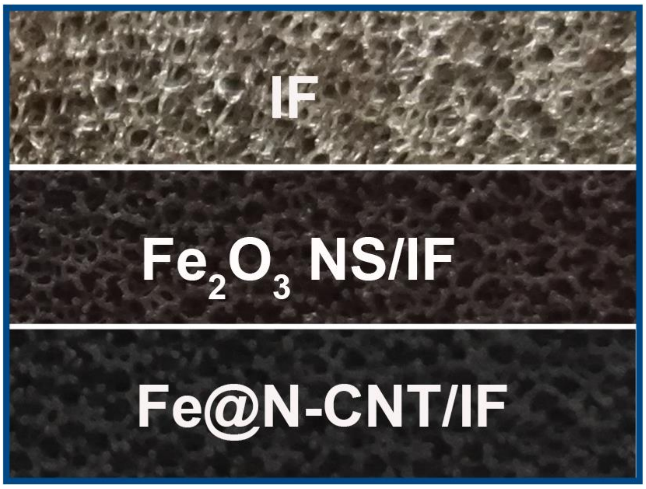

Figure S2 Optical images of IF, Fe_2O_3 NS/IF and Fe@N-CNT/IF.

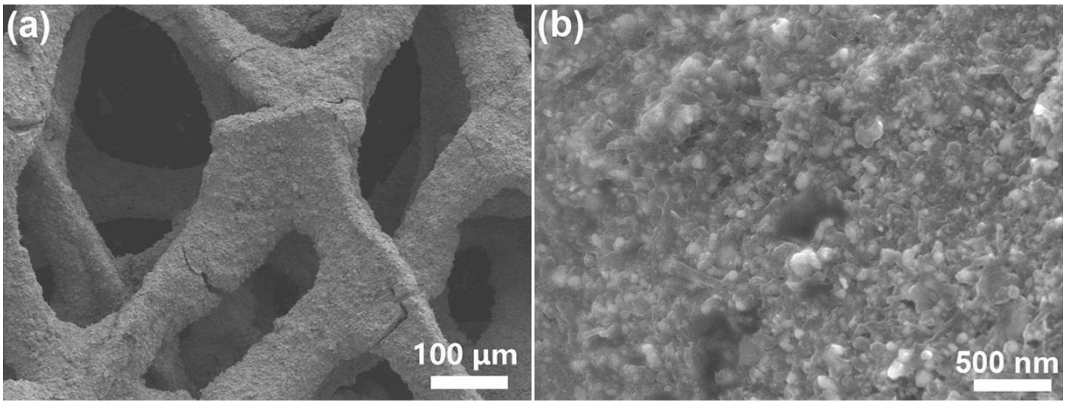

Figure S3 SEM images of Fe@N-C/IF.

A piece of IF and cyanamide were put at a porcelain boat with cover. The weight ratio for IF to dicyandiamide was 1:10. The sample was heated at 450 ℃ for 2 h, then at 750 ℃ for 2 h under Ar atmosphere. The black product was denoted as Fe@N-C/IF. **Figure S3** showed the field-emission scanning electron microscopy (FESEM) image of Fe@N-C/IF with rough surface, however, there was no carbon nanotube observed on the surface.

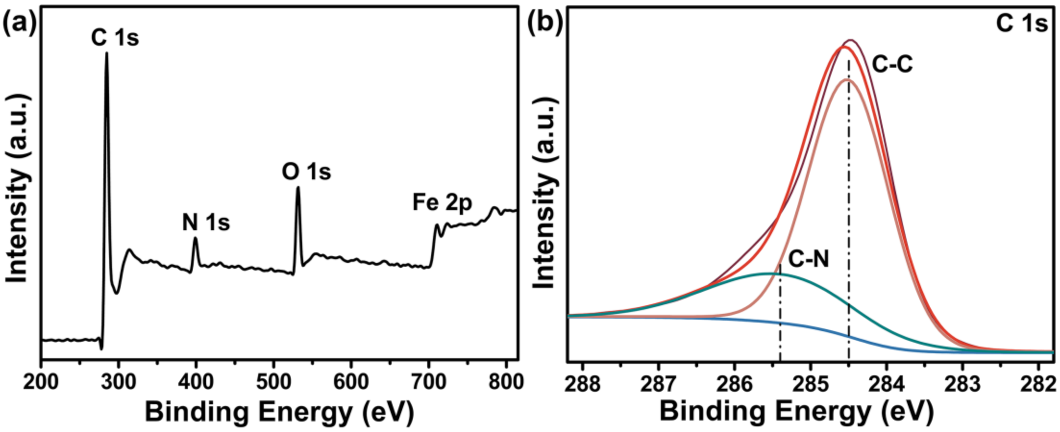

Figure S4 XPS spectra in the (a) full spectrum and (b) C 1s regions for Fe@N-CNT/IF.

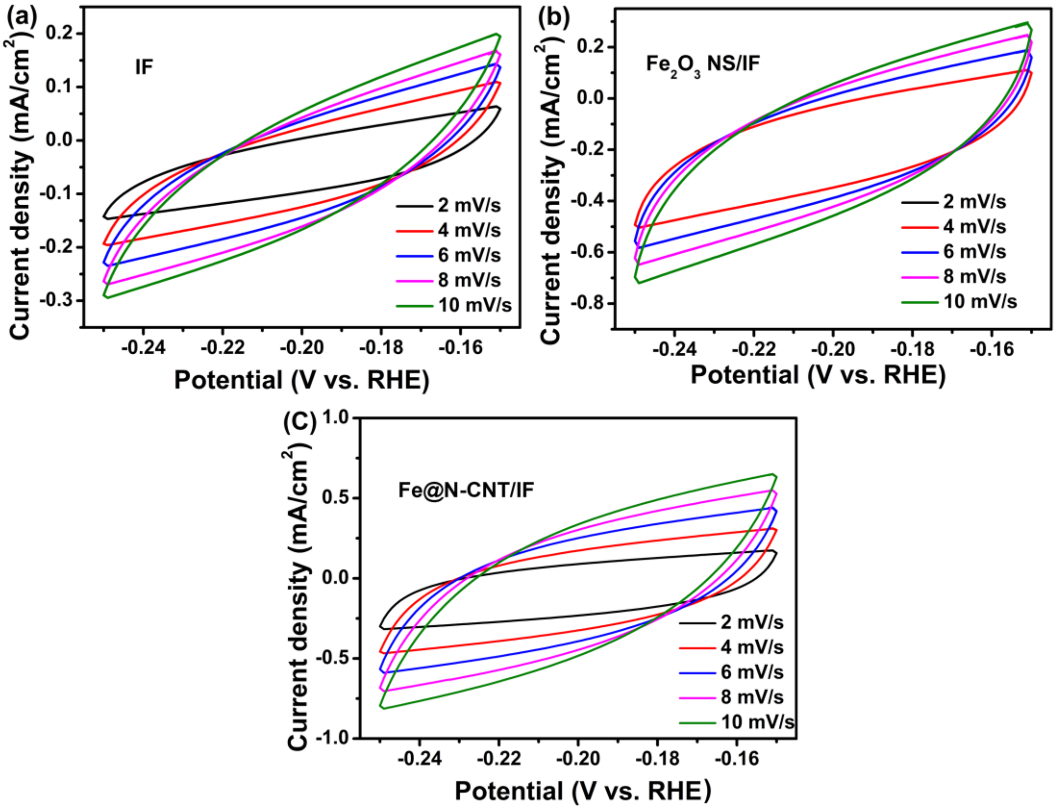

Figure S5 Cyclic voltammograms without faradaic reactions of (a) IF, (b) Fe_2O_3 NS/IF and (c) Fe@N-CNT/IF within the range of -0.15 to -0.25 V.

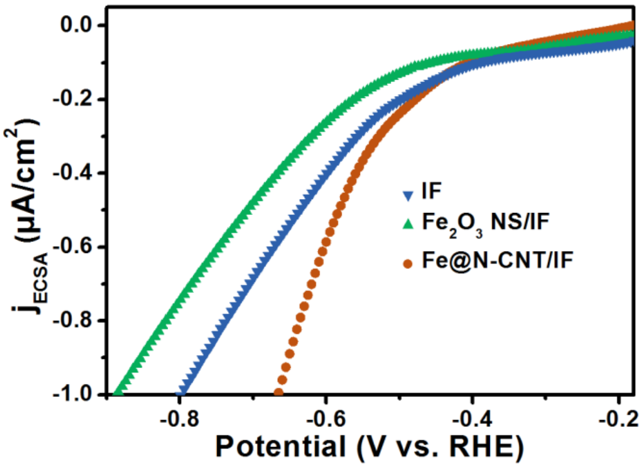

Figure S6 Polarization curves of IF, Fe_2O_3 NS/IF and Fe@N-CNT/IF normalized by the respective electrochemical surface area.

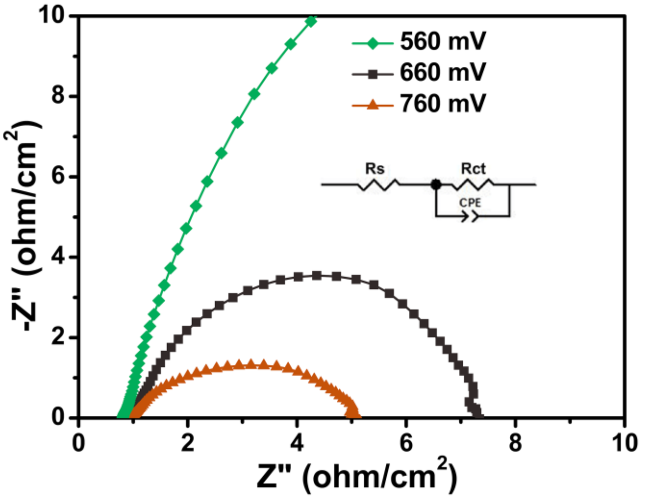

Figure S7 Nyquist plots of Fe@N-CNT/IF at various overpotentials.

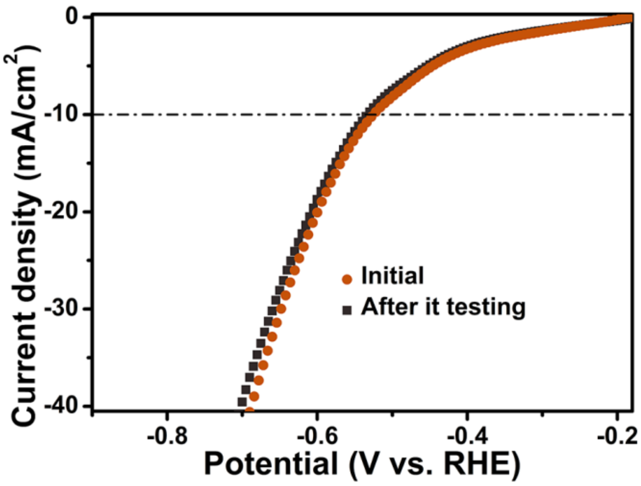

Figure S8 The polarization curves of Fe@N-CNT/IF before and after i-t testing for 10 h.

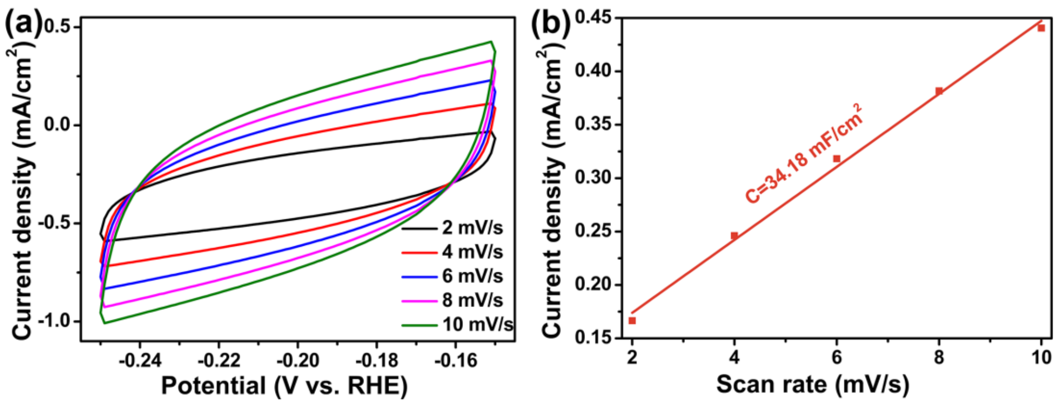

Figure S9 (a) Cyclic voltammograms without faradaic reactions within the range of -0.15 to -0.25 V and (b) the double-layer charging currents at -0.2 V vs. RHE as a function of scan rate of Fe@N-CNT/IF after i-t testing for 10 h.

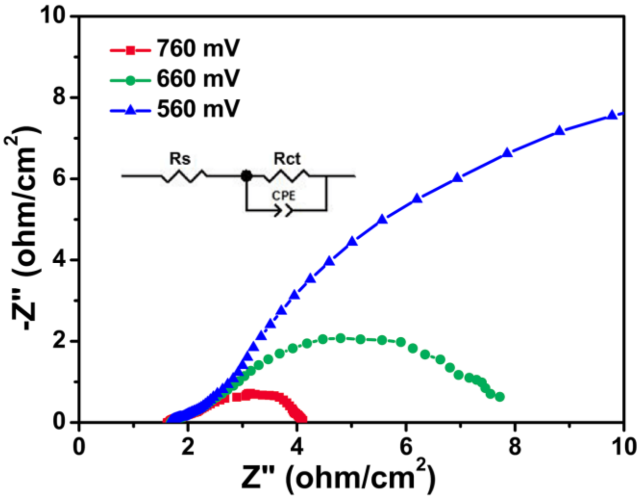

Figure S10 Nyquist plots of Fe@N-CNT/IF at various overpotentials after i-t testing for 10 h.

.

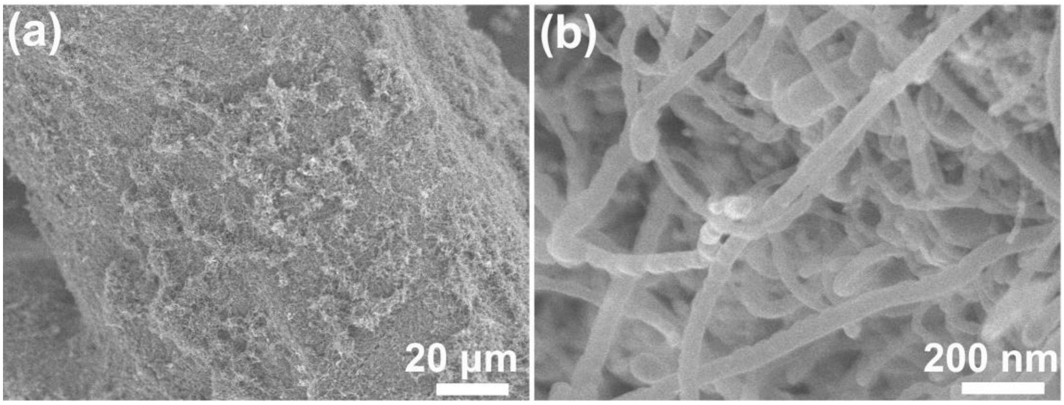

Figure S11 SEM images of Fe@N-CNT/IF after it testing.

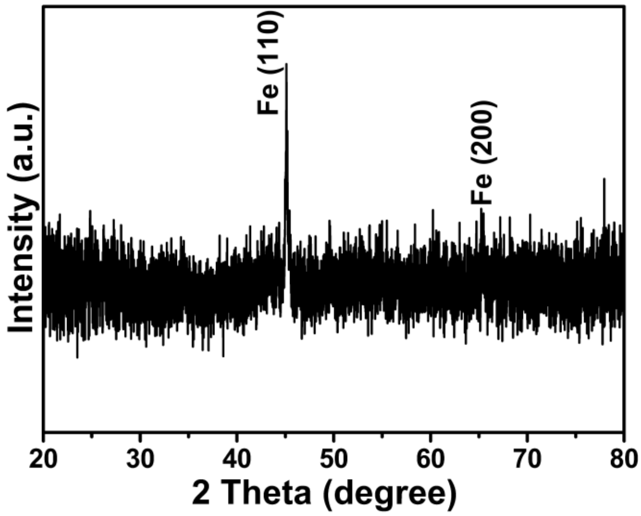

Figure S12 XRD pattern of Fe@N-CNT/IF after it testing.

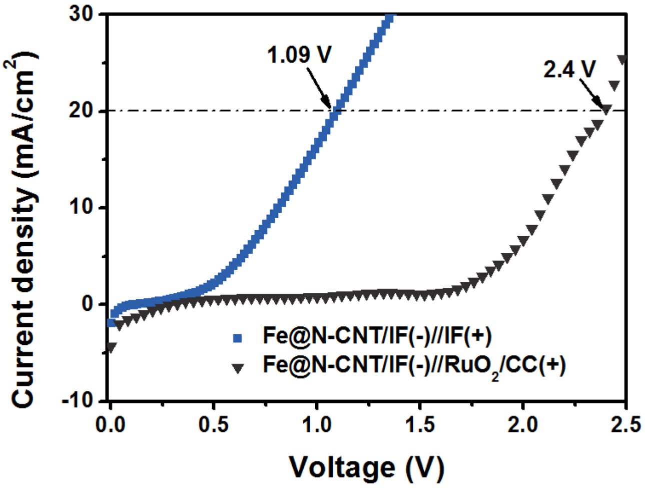

Figure S13 Polarization curves of Fe@N-CNT/IF(-)//IF(+) and Fe@N-CNT/IF(-)//RuO_2/CC in 0.5 M Na_2SO_4.

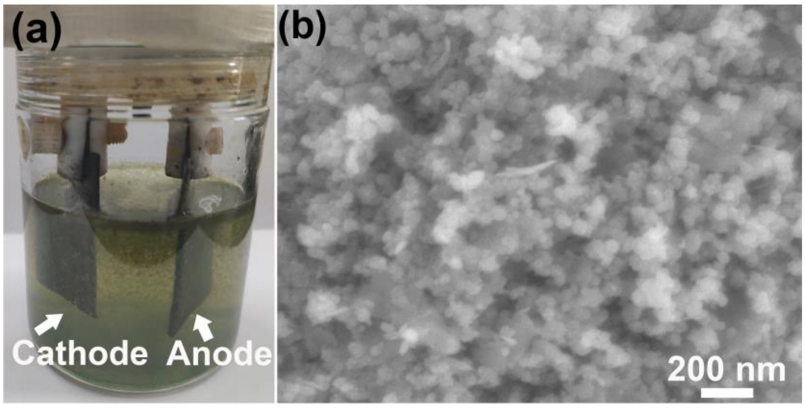

Figure S14 (a) Optical image of Fe@N-CNT/IF(-)//IF(+) at the voltage of 1.5 Vand (b) SEM image of flocculant.

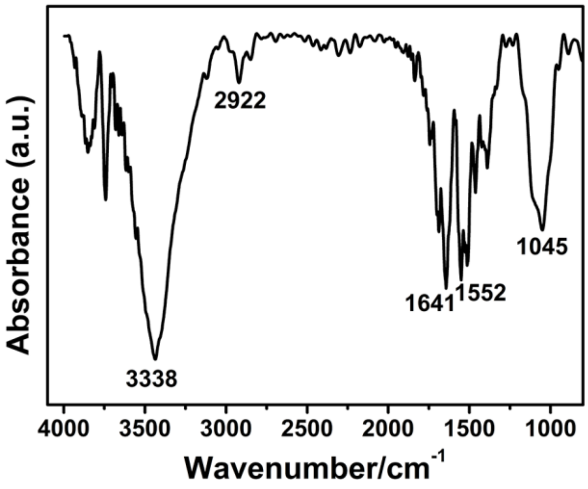

Figure 15 FT-IR spectrum of flocculant.

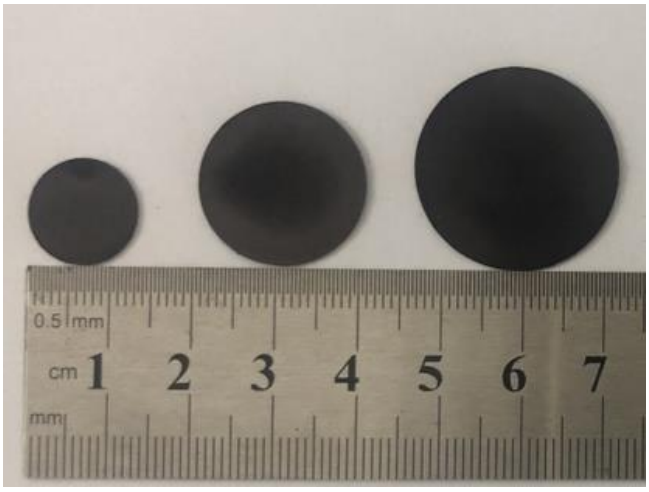

Figure S16 The photos of Fe-C electrodes before calcination.

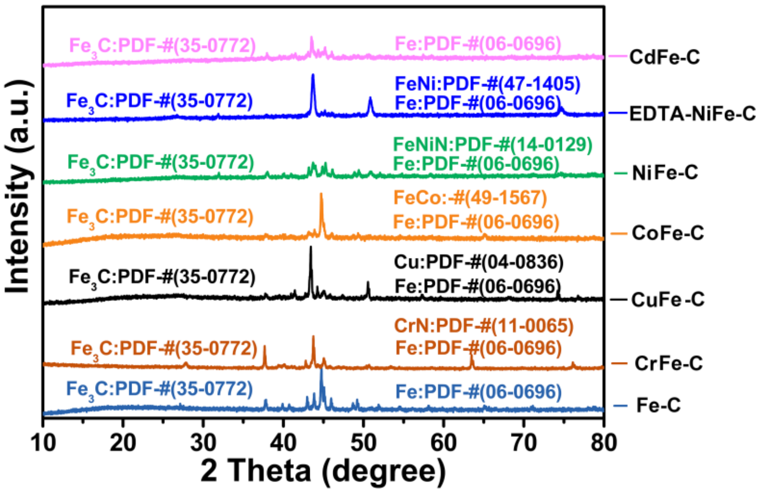

Figure S17 XRD patterns of FeCu-C, FeNi-C, FeCr-C, FeCo-C, FeEDTA-Ni-C and FeCd-C.

The X-ray diffraction (XRD) pattern was used to identify the crystal structure of Fe-C and MFe-C (M=Cr, Ni, Co, Cu, EDTA-Ni, Cd) as shown in **Figure S17**. It is clearly show that all of the samples existed of the diffraction peaks belong to crystal planes of Fe (JCPDS No. 06-0696) and Fe_3C (JCPDS No. 35-0772). In addition, other components such CrN for CrFe-C, Cu for CuFe-C, FeCo for CoFe-C, FeNiN for NiFe-C, FeNi for ENTA-NiFe-C were detected.

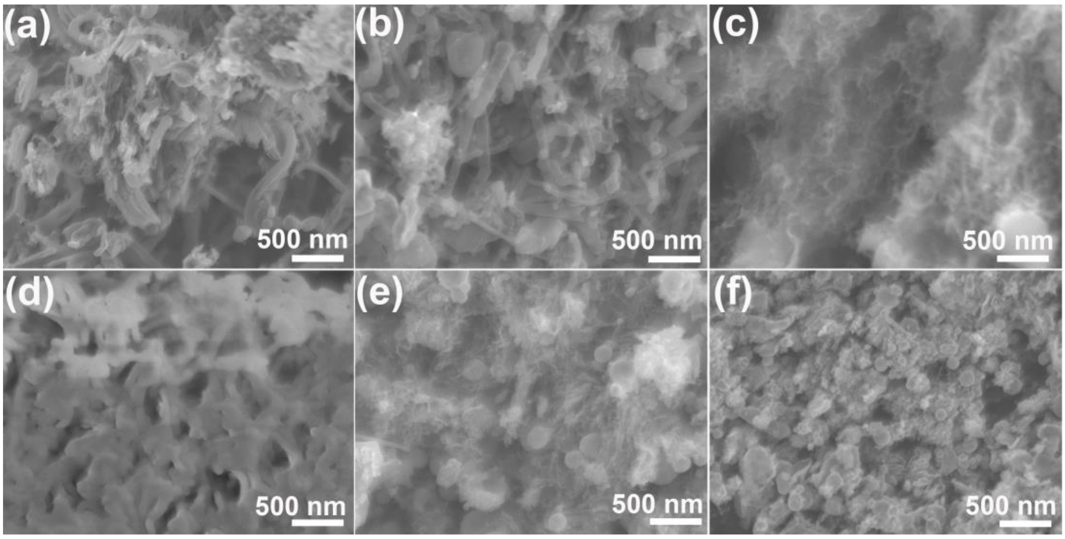

Figure S18 FESEM images for (a) CuFe-C, (b) NiFe-C, (c) CrFe-C, (d) CoFe-C, (e) EDTA-NiFe-C, (f) CdFe-C.

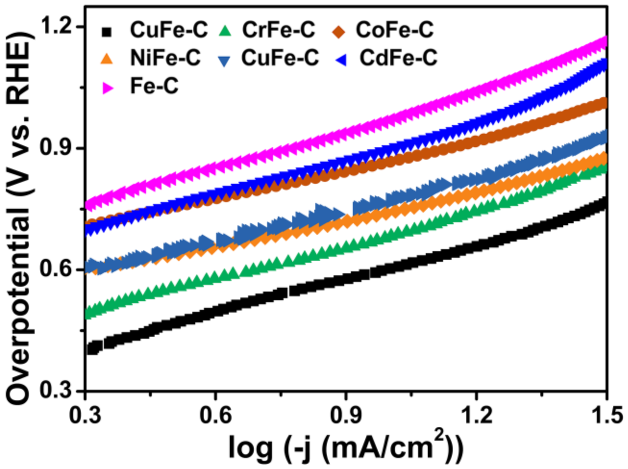

Figure S19 Corresponding Tafel plots (overpotential versus log current density) derived from Figure 4a.

Evaluated the economy of this hybridization system:

$A_1=\Delta U\cdot I\cdot t\cdot P_1$

where A_1, ΔU, I, t and P_1 are the cost, voltage, current, time and univalence of electricity, respectively.

$A_2= D_{Fe}\cdot t\cdot P_2$

where A_2, D_{Fe}, t and P_2 are the cost, the dissolution rate of Fe voltage, time and univalence of Fe, respectively.

$A=A_1-A_2$

where A is the cost of the hybridization system.

Table S1 Comparison of HER performance in acid/neutral/alkaline media for Fe@N-CNT/IF with other non-noble HER electrocatalysts.

Catalysts	Electrolytes	Overpotential@j ($mV@mA\ cm^{-2}$)	Catalyst loading ($mg\ cm^{-2}$)	Reference
Fe@N-CNT/IF	0.5 M H_2SO_4	108@10	1.2	This work
	1.0 M PBS	130@10		
	1.0 M KOH	153@10		
FeP NPs@NPC	0.5 M H_2SO_4	130@10	1.4	Nanoscale 2017, 9, 3555
	1.0 M PBS	386@10		
	1.0 M KOH	241@10		
NiSA-MoS_2	0.5 M H_2SO_4	110@10	-	Nano Energy, 2018, 53, 458
	1.0 M KOH	98@10		
Ni/β-Mo_2C	0.5 M H_2SO_4	155@10	0.14	Chem. Commun. 2018, 54, 9901
	1.0 M PBS	149@10		
	1.0 M KOH	157@10		
Co-NCNT/CC	0.5 M H_2SO_4	78@10	3.4	ChemSusChem 2015, 8, 1850
	1.0 M PBS	170@10		
	1.0 M KOH	180@10		
CoNC/GD	0.5 M H_2SO_4	340@10		ACS Appl. Mater. Interfaces 2016, 8, 31083
	1.0 M PBS	368@10	-	
	1.0 M KOH	284@10		
WON@NC NAs/CC	0.5 M H_2SO_4	106@10	7.7	ChemSusChem 2015, 8, 2487
	1.0 M PBS	152@10		
	1.0 M KOH	130@10		

Mo_2C QD/NGCL	0.5 M H_2SO_4	136@10	1.0	Chem. Commun. 2016, 52, 12753
	1.0 M PBS	136@10		
	1.0 M KOH	111@10		
Co-Ni-B	0.1 M $HClO_4$	209@10	2.1	Appl. Catal. B: Environ. 2016, 192, 126
	0.5 M KPi	170@10		
	1.0 M NaOH	133@10		
CNF@CoS_2	0.5 M H_2SO_4	110@10	6.6	Inorg. Chem. Front. 2016, 3, 1280
	1.0 M PBS	360@10		
	1.0 M KOH	207@10		
Co-C-N	0.5 M H_2SO_4	138@10		J. Am. Chem. Soc. 2015, 137, 15070
	1.0 M PBS	276@10	-	
	1.0 M KOH	178@10		
Co-NRCNTs	0.5 M H_2SO_4	260@10	0.28	Angew. Chem., Int. Ed. 2014, 126, 4461
	1.0 M PBS	540@10		
	1.0 M KOH	370@10		

Table S2 Comparison of the reported electrolyzers.

**Electrode**	**Electrolyte**	**Reaction type**	**j (mA/cm^2)**	**Potential (V)**	**Reference**
**Fe@N-CNT/IF (-)//IF(+)**	0.5 M Na_2SO_4	HER and Electroflocculation	20	1.09	This work
**Ni_3S_2/Ni(-) //Ni_3S_2/Ni(+)**	1.0 M KOH	HER and Oxidative Biomass	20	1.52	J. Am. Chem. Soc. 2016, 138, 13639
**NiCoP(-)//NiCoP (+)**	1.0 M KOH	Overall water splitting	10	1.58	Nano Lett. 2016, 16, 12, 7718
**Ni_2P NPA/NF(-)// Ni_2P NPA/NF(+)**	1.0 M KOH	HER and Oxidative Biomass	10	1.44	Angew. Chem. Int. Ed. 2016, 55, 9913
**hp-N(-)//hp-N(+)**	1.0 M KOH	HER and	50	~1.40	ACS Catal. 2017,

		Oxidative Alcohols			7, 4564
**$NiCo_2O_4$(-)// $NiCo_2O_4$ (+)**	1.0 M KOH	Overall water splitting	20	1.65	Angew. Chem. Int. Ed. 2016, 128, 6398
**NiFe LDH/NF (-)//NiFe LDH/NF(+)**	1.0 M KOH	Overall water splitting	10	1.7	Science 2014, 345, 1593
**Ni_5P_4/NF(-)// Ni_5P_4/NF(+)**	1.0 M KOH	Overall water splitting	10	1.7	Angew. Chem. Int. Ed. 2015, 54, 12361
**Co-P(-)//Co-P(+)**	1.0 M KOH	Overall water splitting	10	1.64	Angew. Chem. Int. Ed. 2015, 54, 6251
**S-MnO_2/NF(-)// S-MnO_2 (+)**	1.0 M KOH	HER and Oxidative Urea	10	1.41	Angew. Chem. Int. Ed. 2016, 55, 3804
**Fe_2P/SSM(-)// Fe_2P/SSM(+)**	1.0 M KOH	HER and Oxidative Glucose	10	1.52	Electrochem.Commun., 2017, 83, 11
**$Ni_{0.51}Co_{0.49}P$(-)// $Ni_{0.51}Co_{0.49}P$ (+)**	1.0 M KOH	Overall water splitting	10	1.57	Adv. Funct. Mater., 2016,26, 42, 7644

[1] R. Lei, H. Ni, R. Chen, B. Zhang, W. Zhan, Y. Li, *J. Mater. Sci.: Mater. Electron.*, **2017**, 28, 10481.
